# β-Glucan-Induced Immuno-Modulation: A Role for the Intestinal Microbiota and Short-Chain Fatty Acids in Common Carp

**DOI:** 10.3389/fimmu.2021.761820

**Published:** 2022-01-06

**Authors:** Jules Petit, Irene de Bruijn, Mark R. G. Goldman, Erik van den Brink, Wilbert F. Pellikaan, Maria Forlenza, Geert F. Wiegertjes

**Affiliations:** ^1^ Aquaculture and Fisheries Group, Department of Animal Sciences, Wageningen University & Research, Wageningen, Netherlands; ^2^ Department of Microbial Ecology, Netherlands Institute of Ecology-The Royal Netherlands Academy of Arts and Sciences, (NIOO-KNAW), Wageningen, Netherlands; ^3^ Animal Nutrition Group, Department of Animal Sciences, Wageningen University & Research, Wageningen, Netherlands

**Keywords:** SCFA, immunomodulation, fish, β-glucan, microbiota

## Abstract

Dietary supplementation of fish with β-glucans has been commonly associated with immunomodulation and generally accepted as beneficial for fish health. However, to date the exact mechanisms of immunomodulation by β-glucan supplementation in fish have remained elusive. In mammals, a clear relation between high-fibre diets, such as those including β-glucans, and diet-induced immunomodulation *via* intestinal microbiota and associated metabolites has been observed. In this study, first we describe by 16S rRNA sequencing the active naive microbiota of common carp intestine. Based on the abundance of the genus *Bacteroides*, well known for their capacity to degrade and ferment carbohydrates, we hypothesize that common carp intestinal microbiota could ferment dietary β-glucans. Indeed, two different β-glucan preparations (curdlan and MacroGard^®^) were both fermented *in vitro*, albeit with distinct fermentation dynamics and distinct production of short-chain fatty acids (SCFA). Second, we describe the potential immunomodulatory effects of the three dominant SCFAs (acetate, butyrate, and propionate) on head kidney leukocytes, showing effects on both nitric oxide production and expression of several cytokines (*il-1b*, *il-6*, *tnfα*, and *il-10*) *in vitro*. Interestingly, we also observed a regulation of expression of several *gpr40L* genes, which were recently described as putative SCFA receptors. Third, we describe how a single *in vivo* oral gavage of carp with MacroGard^®^ modulated simultaneously, the expression of several pro-inflammatory genes (*il-1b*, *il-6*, *tnfα*), type I IFN-associated genes (*tlr3.1*, *mx3*), and three specific *gpr40L* genes. The *in vivo* observations provide indirect support to our *in vitro* data and the possible role of SCFAs in β-glucan-induced immunomodulation. We discuss how β-glucan-induced immunomodulatory effects can be explained, at least in part, by fermentation of MacroGard^®^ by specific bacteria, part of the naive microbiota of common carp intestine, and how a subsequent production of SFCAs could possibly explain immunomodulation by β-glucan *via* SCFA receptors present on leukocytes.

## Introduction

Effects of immunomodulation by β-glucans have been widely studied in teleost fish. Regardless of administration route or fish species, β-glucans are often associated with immunomodulatory effects and increased resistance to both viral and bacterial infections [as reviewed by (1–3)]. Although supplementation of β-glucans may have become common practise in aquaculture, modulation routes and mechanisms explaining the observed immunomodulatory effects of β-glucans in the fish intestine have remained largely obscure.

In mammalian vertebrates, direct recognition of β-glucans is mediated by at least the C-type lectin receptor (CLR) Dectin-1 (4–6), whereas in invertebrates β-glucans can be recognised by β-glucan-binding proteins (7). For both mammals (8, 9) and invertebrates (7, 10), several non-exclusive pathways play a downstream role in the response to β-glucans, often mediating activation of the transcription factor NF-κB (11). Although in vertebrate fish genomes no clear homologue of mammalian Dectin-1 could be identified so far (6, 12), we could show activation of carp macrophages with curdlan (13, 14), considered a Dectin-1-specific (1,3)-β-glucan stimulus. We therefore proposed that the immuno-modulatory effects of β-glucan in carp macrophages could include regulation of a downstream signalling pathway typical of CLR activation and confirmed our hypothesis by pathway analysis of differentially expressed genes (DEGs) (14). Among the regulated genes were *card9* and *bcl10*, both members of the signalling complex downstream of mammalian Dectin-1. Not only did we observe a regulation of the CLR signalling pathway, several candidate receptors could be identified based on regulation of expression upon stimulation with β-glucans, or based on the conservation of β-glucan-binding motifs in their predicted translations to protein (14). As there is clear evidence for presence of macrophages and other antigen-sampling cells in the fish intestine [as reviewed by (15)], it is possible that recognition of β-glucans in the intestine occurs *via* a direct recognition mechanism of β-glucan receptors.

Another mechanism of immune modulation by β-glucans could be indirect, relying on prebiotic effects inducing a shift in intestinal microbiota which would influence the microenvironment of the intestine. The two mechanisms explaining immunomodulation by β-glucans (direct recognition and indirect prebiotic effects) should not necessarily be exclusive. The intestinal microbiota is of well-known importance to the host, for example *via* degradation and fermentation of non-starch polysaccharides and/or resultant production of metabolites, which in turn affect the host immune system [as reviewed by (16–20)]. Also for fish, the importance of microbial communities for their health and the mitigation of diseases has been acknowledged [as reviewed by (19–21)]. Several studies have shown the ability of certain fish microbiota to degrade carbohydrates, showing growth of specific intestinal bacterial monocultures on different carbohydrates, among which is β-glucan (22), and observing the *in vitro* fermentation of several carbohydrates and production of short-chain fatty acids (SCFAs) by intestinal microbes of common carp (*Cyprinus carpio*) and Nile tilapia (*Oreochromis niloticus*) (23, 24). In a comparison of the fermentability of different carbohydrates and produced SCFAs between Nile tilapia and European sea bass, both their gut microbiota were found capable of fermenting carbohydrates but with differing end-product profiles (25). Recently, a systematic review extensively described the capacity of tilapia to digest carbohydrates (26). Still, documentation on the effects of dietary β-glucan supplementation on the composition of the intestinal microbiota is relatively scarce. In common carp, a 2-week feeding with β-glucan-enriched diets induced shifts in the intestinal microbiota, manifested either as a reduction in species richness and absolute number of operational taxonomic units (OTUs) (27) or as an increase in microbial diversity (28). In seabass, a 4-week feeding with β-glucan-enriched diets induced a shift toward more *Methylobacterium* (29), whereas in rainbow trout and feral common carp, an 8-week feeding with yeast-derived probiotics rich in β-glucans induced a shift toward more lactic acid bacteria (30, 31). Recently, a study investigating the effect of feeding β-glucans for 7 weeks to common carp showed, through DGGE analysis, a possible shift in bacterial species richness alongside a downregulation of *il-1b* expression (32). Also, addition of β-glucans to the water could affect the composition of intestinal microbiota, at least of Nile tilapia (33). Overall, possibly owing to differences in diet formulation, sampling, microbial analysis, and fish species, no consistent and distinct shift in intestinal microbial composition induced by β-glucans has been discerned. This leaves undecided if immunomodulation by β-glucans is based on a) receptor-mediated recognition or b) fermentation of β-glucans facilitating a subsequent shift in composition of intestinal microbiota with an associated difference in SCFA metabolic profile, or both.

In the present study, we investigated the role of β-glucan as prebiotic first based on the hypothesis that common carp intestinal microbiota would be able to ferment β-glucans. We further hypothesised that, as a result of this fermentation process, *in vivo* treatment of carp with β-glucans would induce a shift in intestinal microbial composition and subsequently might result in a shift in production of short-chain fatty acid (SCFA) metabolites. Finally, we hypothesised that this series of processes would contribute to the commonly observed immunomodulation by β-glucans of the host. To test our hypotheses, we first characterised the naive, active intestinal microbiota of common carp by 16S rRNA transcript sequencing. We also analysed the SCFA profile generated during *in vitro* fermentation and noticed β-glucan-dependent shifts in SCFA production. To this purpose, we used an *in vitro* batch culture method to assess the potential fermentability of feed ingredients (25, 34, 35). With this method, an inoculum is prepared under strictly anaerobic conditions using freshly collected intestinal contents. The inoculum is incubated with a fermentable substrate of choice and with a medium to support bacterial growth. Subsequently, gas accumulating during fermentation indicates the kinetics of fermentation. At the end of the fermentation period, samples are taken from the fermentation fluid in order to analyse fermentation end-products and substrate utilization (36). Subsequently, we performed further *in vitro* studies into the modulation by SCFAs of leukocyte function and possible involvement of G-protein-coupled receptor (GPR) 40 family members. Our recent study of phylogeny and synteny of several cDNA sequences for carp *gpr40L* genes (37) showed a division into three subclasses (a, b, and c), all structurally conserved. Here, we present a first investigation of the importance of these putative SCFA receptors in the route to immunomodulation by β-glucans after microbial fermentation by studying the regulation of *gpr40L* gene expression. Last but not least, common carp were treated with a single oral gavage with β-glucans to analyse the *in vivo* effects of β-glucans on intestinal microbiota and effects on regulation of local gene expression. Overall, our data suggest that dietary β-glucan administration may have indirect immunomodulatory effects, associated with a shift in SCFA profile and detection of these SCFAs by *gpr40L* SCFA receptors. The interconnection between SCFA profile shifts and detection of such by the appropriate receptors warrants further investigation.

## Material and Methods

### Animals

European common carp (*Cyprinus carpio carpio* L.) of the R3 × R8 strain were used, which originated from a cross between the Hungarian R8 strain and the Polish R3 strain (38). Carp were bred and raised in the aquatic research facility of Wageningen University at 24°C in recirculating UV-treated water and fed pelleted dry food (Skretting, Nutreco) twice daily. Fish were housed in rectangular 60-l tanks connected to the same recirculation system, resulting in common water supply. All experiments were performed with the approval of the animal experiment committee of Wageningen University (DEC number 2015098).

### Collection of Intestinal Contents for 16s rRNA Profiling

Nine-month-old fish (20–40 g, n = 5) were starved for 24 h and subsequently euthanised in 0.3 g/l tricaine methane sulfonate (TMS) (Crescent Research Chemicals, Phoenix, USA) in aquarium water buffered with 0.6 g/l sodium bicarbonate and bled *via* the caudal vein. The third segment of the intestine was isolated, as this segment shows a stable microbiota composition (39), and intestinal content of these samples was collected by scraping. Samples were snap-frozen in liquid nitrogen and stored at -80°C.

### Total Bacterial RNA Isolation

Prior to isolation of RNA from intestinal content, all samples were weighed. Total RNA from intestinal content was isolated using the RNeasy PowerMicrobiome Kit (Qiagen, 26000-50), following the manufacturer’s instructions. Agarose gel electrophoresis and a ND1000 spectrophotometer (NanoDrop Technologies, Wilmington, DE, USA) were used to control RNA yield and quality (260/280: >2.0, 260/230: >2.0). RNA samples were stored at -80°C until further use.

### 16S rRNA Profiling

Total community analyses were performed on initially five replicates per treatment similar as described for Perez-Jaramillo et al. (40). Briefly, total community RNA was used for amplification and sequencing of the 16S rRNA, targeting the variable V3–V4 regions resulting in amplicons of approximately ~460 bp. Illumina 16S rRNA gene amplicon libraries were generated and sequenced at BaseClear (Leiden, the Netherlands). In short, barcoded amplicons from the V3–V4 region of 16S rRNA genes were generated using a 2-step approach. Ten ng RNA was used as template for the One-Step RT-PCR Kit (Qiagen^®^) employed according to the manufacturer’s instructions with a total volume of 50 μl using the 341F (5′-CCTACGGGNGGCWGCAG-3′) and the 785R (5′-GACTACHVGGGTATCTAATCC-3′) primers appended with Illumina adaptor sequences. Control PCR reactions were performed alongside each separate amplification without addition of template. PCR products were purified using AMPure XP beads according to the manufacturer’s instructions, and the sizes of the PCR products were checked on a Fragment Analyzer (Advanced Analytical) and quantified by fluorometric analysis. Purified PCR products were used for the 2nd PCR in combination with sample-specific barcoded primers (Nextera XT Index Kit, Illumina). Subsequently, PCR products were purified, checked on a Fragment Analyzer (Advanced Analytical), and quantified, followed by multiplexing, clustering, and sequencing on an Illumina MiSeq with the paired-end (2×) 300-bp protocol and indexing. For each independent sample, at least 25,000 raw reads were sequenced. The sequencing run was analysed with the Illumina CASAVA pipeline (v1.8.3) with demultiplexing based on sample-specific barcodes. The raw sequencing data produced were processed removing the sequence reads of too low quality (based on “*failed-chastity<=1*”, using a chastity threshold of 0.6, on the first 25 cycles) and discarding reads containing adaptor sequences or PhiX control with an in-house filtering protocol. A quality assessment on the remaining reads was performed using the FASTQC quality control tool version 0.10.0.

### Bacterial Community Analyses

The RDP extension to PANDASeq (41) named Assembler (42) was used to merge paired-end reads with a minimum overlap of 10 bp and at least a PHRED score of 25. Primer sequences were removed from the paired sample FASTQ files using FLEXBAR version 2.5 (43). Sequences were converted to FASTA format and concatenated into a single file. All reads were clustered at 97% similarity into OTUs using the UPARSE strategy by dereplication, sorting by abundance with at least two sequences and clustering using the UCLUST smallmem algorithm (44). These steps were performed with VSEARCH version 1.0.10 (45), which is an open-source and 64-bit multithreaded compatible alternative to USEARCH. Next, chimeric sequences were detected using the UCHIME algorithm (46) implemented in VSEARCH. All reads before the dereplication step were mapped to OTUs using the usearch_global method implemented in VSEARCH to create an OTU table and converted to BIOM-Format 1.3.1 (47). Finally, taxonomic information for each OTU was added to the BIOM file by using the RDP Classifier version 2.10 (48). Representative OTU sequences were assigned to a taxonomic classification *via* BLAST against the Silva database (version 128). All steps were implemented in a Snakemake workflow (49), which is available on GitHub^1^. For downstream analysis, we took the obtained OTU table and prepared a “filtered table” using QIIME (1.9.1) custom scripts (50). First, we extracted from the OTU table the Bacteria domain using the command *split_otu_table_by_taxonomy.py*. Next, we discarded singletons, doubletons, Chloroplast, and Mitochondria sequences using the command *filter_otus_from_otu_table.py*.

### *In Vitro* Gas Production Technique

#### Collection of Intestinal Contents for *In Vitro* Fermentation

Naive carp were euthanised with 0.3 g/l TMS in aquarium water buffered with 0.6 g/l sodium bicarbonate and bled *via* the caudal vein. Subsequently, fish were put on ice and the intestine, without bulbus, was removed. The content of the intestine was collected in pre-weighed plastic tubes that were filled with CO_2_ to ensure anaerobic conditions. The intestinal content of ten fish was pooled, and five independent pools were used for subsequent *in vitro* fermentation.

#### Substrates for *In Vitro* Fermentation

Four different substrates were used for *in vitro* gas production analyses: glucose, (D-glucose monohydrate; Merck, Darmstadt, Germany), PBS (cell culture grade, Lonza), curdlan (β-(1,3)-glucan extracted from *Alcaligenes faecalis*, Sigma-Aldrich), and MacroGard® [a cell wall preparation of *S. cerevisiae* comprising at least 60% β-(1,3/1,6)-glucan (Zilor, São Paulo, Brazil)]. Glucose was included as a readily fermentable substrate for comparative purposes (25).

#### Inoculum Preparation and Measurement of Cumulative Gas Production

Pooled intestinal content was immediately transported to the laboratory after collection where it was weighed and transferred to a beaker. The contents of the beaker were stirred and flushed with a constant gentle stream of CO_2_. Pre-warmed (25°C) anaerobic, sterile saline (9 g/l NaCl) was added in a ratio of 1:5 (W/V) to ensure sufficient amount of inoculate. The diluted material was homogenised using a vortex mixer and strained through a double layer of cheese cloth with 16 threads per cm in both directions. From the resulting inoculate, 5 ml was then dispensed into a pre-warmed 300-ml fermentation bottle, containing 0.5 g substrate and 82 ml of medium. Three replicate bottles for each substrate per inoculate were used. The medium consisted of a basal solution containing micronutrients required by the microbial population for growth, a bicarbonate solution, a vitamin/phosphate solution, and a reducing agent. The composition of the medium is described in detail by Williams et al. (51). Subsequently, bottles were immediately attached to an automated gas production system (52). Within this system, pressure sensors detect a fixed pressure, after which a computer software program allows opening of a valve to release gas, the time at which this occurred was recorded. Bottles were incubated for 168 h at 25°C, equal to the body temperature of carp.

#### Curve Fitting and Statistics of Cumulative Gas Production

A monophasic model as described by Groot et al. (53), was fitted to the profile of the cumulative gas production of each fermentation bottle according to the equation G = A/(1 + (C/t)B), where G is the total millilitre gas produced per gram organic matter (OM) at time t; A is the asymptotic gas production (mL/g OM); B is the switching characteristic of the curve; C is the half time (time at which half of the asymptote is reached); and t is the time (h).

The maximum rate of gas production (Rmax) and the time at which it occurs (Tmax) were calculated according to (54) as


$$R_{\max}=(A(C^{B})B(T_{\max}{(-B-}^{1)})/{\left(1+({\text{C}}^{B})(T_{\max}{(-B}^{)})\right)}^{2}\text{ and }T_{\max}=C(((B–\;1)/{\left(B+1\right)}^{\left(1/B\right)})$$

For each bottle, curve fitting was done using the non-linear least-square regression procedure NLIN (SAS Inst. Inc., Cary, NC, USA). One-way analysis of variance using the GLM procedure of SAS (SAS Inst. Inc.) was used to test the effect of substrate on gas production parameters and fermentation end products. The average of replicate bottles per substrate per inoculum was considered as the experimental unit. The effect of replicate bottles was tested separately and was not significant for any of the parameters. It was therefore excluded from the model and thus became part of the error term.

#### Sampling and Analyses of Fermentation Liquid

At the end of the incubation period, pH of the fermentation fluids was recorded and samples were collected for analysis of ammonia (NH_3_), SCFAs, and lactate. Analyses of NH_3_ and SCFA were determined as described previously (55), using gas chromatography for SCFA analysis (GC; Fisons HRGC Mega 2, CE Instruments, Milan, Italy). Lactate was measured using the Lactate Colorimetric Assay Kit II (K627, BioVision) according to the manufacturer’s instructions, including the optional filtration step with Amicon 10K spin columns (Z677108-96EA, Sigma-Aldrich, centrifugation for 20 min at 21.100× *g*), as described previously (56).

### Head Kidney Leukocyte Isolation

Carp were euthanised with 0.3 g/l TMS (Crescent Research Chemicals) in aquarium water buffered with 0.6 g/l sodium bicarbonate and bled using vacuettes (BD Vacutainer SST with 21-G needles, Becton Dickinson) *via* the caudal vein. The head kidney was isolated, and total head kidney leukocytes (HKLs) were separated on a 1.02–1.08-g/ml Percoll (GE Healthcare, Thermo Fisher Scientific) density gradient, as previously described (13).

### Nitrogen Radical Production

Production of NO was determined as nitrite accumulation using the Griess reaction, as previously described (57). HKLs were seeded at a density of 1 × 10^6^ per well in 96-well culture plates (CORN3596; Corning) and stimulated with one of the following: RPMI (control) or LPS (L2880, 30 µg/ml, Sigma-Aldrich) in the presence or absence of different concentrations of sodium acetate (0.5–25 mM, S2889-250G, Sigma-Aldrich), sodium butyrate (0.05–25 mM, 303410-100G, Sigma-Aldrich), or sodium propionate (0.05–25 mM, P1880-100G, Sigma-Aldrich) or in the presence or absence of a mix of acetate (62.5%), butyrate (10%), or propionate (27.5%) in the proportion of produced SCFAs after *in vitro* fermentation of MacroGard^®^ at 0.1–25 mM. After 96 h at 27°C in the presence of 5% CO_2_, nitrite production was measured at OD540, using a FilterMax F5 Multi-Mode Microplate Reader and quantified using a sodium nitrite (NaNO_2_) standard curve.

### Reactive Oxygen Species (ROS) Production

Production of reactive oxygen species (ROS) was determined by a real-time luminol-based ECL assay, as previously described (58). HKLs were seeded at a density of 1 × 10^6^ per well in white 96-well plates (CLS3912; Corning) and stimulated with one of the following: RPMI (control), zymosan (tlrl-zyd, 50 µg/ml, InvivoGen) in the presence or absence of different concentrations of sodium acetate (0.5 and 2.5 mM, S2889-250G, Sigma-Aldrich), sodium butyrate (0.1–0.5 mM, 303410-100G, Sigma-Aldrich), or sodium-propionate (0.1–0.5 mM, P1880-100G, Sigma-Aldrich) or in the presence or absence of a mix of acetate (62.5%), butyrate (10%), or propionate (27.5%) in the proportion of produced SCFAs after *in vitro* fermentation of MacroGard^®^ at 0.5–5 mM. Stimulation with PMA (P8139, 1 mg/ml, stimulated control; Sigma-Aldrich) was used as a technical control for the assay (data not shown). SCFA concentrations were based on the outcomes of the nitrogen radical production analysis, and thus fewer SCFA concentrations were included the ROS analysis. Chemiluminescence emission was measured in real time (every 2 min for 120 min) with a FilterMax F5 Multi-Mode Microplate Reader at 27°C and expressed as area under the curve, as previously described (56). Fold changes were calculated as the area under the curve of stimulated HKLs relative to unstimulated HKLs (RPMI without zymosan).

### Head Kidney Leukocyte Stimulation for Gene Expression Analysis

For gene expression analysis, HKLs were seeded at a density of 4.5 × 10^6^ per well in a 24-well plates (CORN3524; Corning) and stimulated with RPMI and LPS (L2880, 30 µg/ml, Sigma-Aldrich) in the presence or absence of a mix of acetate (62.5%), butyrate (10%), or propionate (27.5%) in the proportion of produced SCFAs after *in vitro* fermentation of MacroGard^®^ at 2.5 mM or of sodium acetate (1.55 mM, S2889-250G, Sigma-Aldrich), sodium butyrate (0.25 mM, 303410-100G, Sigma-Aldrich), or sodium propionate (0.765 mM, P1880-100G, Sigma-Aldrich); at the relative concentration, each was included in the 2.5-mM SCFA mix. Each stimulation was performed in technical triplicate and in n = 5 independent experiments. At 3 and 6 h post stimulation, 13.5 × 10^6^ cells were lysed in the lysis buffer from the RNeasy Kit (RLT buffer) and stored at -80°C until RNA isolation.

### Oral Gavage and Tissue Sampling

Carp of 9 months (20–40 g, n = 20 per group) were starved overnight and anaesthetised with one-third the killing dose of TMS (i.e., 0.10 g/l TMS in aquarium water buffered with 0.2 g/l sodium bicarbonate) to allow manipulation without killing. Anaesthetised fish received an oral gavage with 100 µl of PBS (n = 20, Lonza, LO BE17-516F) or MacroGard^®^ dissolved in PBS (n = 20, 10 mg/ml, 1 mg per fish) using a 200-μl pipette. Fish feeding (twice-daily) was resumed at 12 h post gavage. At 3, 7, 11, and 14 days post gavage, before morning feeding, n = 5 fish were euthanised in 0.3 g/l TMS in aquarium water buffered with 0.6 g/l sodium bicarbonate and bled *via* the caudal vein (data for 3, 11, and 14 days are not shown). The third segment of the intestine was isolated, and intestinal content of the third segment was collected by scraping, snap-frozen in liquid nitrogen, and stored at -80°C for 16S rRNA sequencing. A tissue sample of the third segment of the intestine was snap-frozen in liquid nitrogen and stored at -80°C for gene expression analysis.

### Total RNA Isolation and cDNA Synthesis

Total RNA from isolated tissue samples (25–50 mg per sample) and cells (13.5 × 10^6^ cells per sample) was isolated using the RNeasy Mini Kit (Qiagen, 74106), including on-column DNase treatment, according to the manufacturer’s instructions, and stored at −80°C. Prior to cDNA synthesis, total RNA was treated with DNase I, Amplification Grade (Invitrogen), and cDNA was synthesised using random primers (300 ng) and Superscript III First-Strand Synthesis for RT-PCR (Invitrogen). cDNA samples were diluted 25× in nuclease-free water prior to real-time quantitative PCR (RT-qPCR) analysis.

### Gene Expression Analysis

Gene expression was measured with RT-qPCR using ABsolute qPCR SYBR Green Mix (Thermo Scientific) in a Rotor-Gene Q (Qiagen) as previously described (59). Fluorescence data were analysed using Rotor-Gene Analysis software version 1.7. The relative expression ratio (R) of each sample was calculated according to the Pfaffl method (60) based on the take-off deviation of sample versus each of the PBS controls and normalised relative to the s11 protein of the 40-s subunit as reference gene (see **Table 1** for primer information).

**Table 1:** Overview of RT-qPCR primers used for in the current study.

Primer	Gene name	Forward (5′–3′)	Reverse (5′–3′)	GenBank accession no.
*40s*	*40S ribosomal protein S11*	CCGTGGGTGACATCGTTACA	TCAGGACATTGAACCTCACTGTCT	AB012087
*cxca*	*CXC chemokine a (interleukin-8)*	GGGTGTAGATCCACGCTGTC	CTTTACAGTGTGGGCTTGGAG	AJ550164
*cxcb*	*CXC chemokine b*	GCTGCCTGCTTGTTGTAGAG	ATCTGTTTTGGAGGAACCA	AB082985
*il-1b*	*Interleukin-1b*	AAGGAGGCCAGTGGCTCTGT	CCTGAAGAAGAGGAGGAGGCTGTCA	AJ245635
*il-6a*	*Interleukin-6a*	CAGATAGCGGACGGAGGGGC	GCGGGTCTCTTCGTGTCTT	KC858890
*il-6b*	*Interleukin-6b*	GGCGTATGAAGGAGCGAAGA	ATCTGACCGATAGAGGAGCG	KC858889
*il-10a*	*Interleukin-10a*	CGCCAGCATAAAGAACTCA	TGCCAAATACTGCTCAATGT	cypCar_00007086,LHQP01030085
*il-10b*	*Interleukin-10b*	CGCCAGCATAAAGAACTCGT	TGCCAAATACTGCTCGATGT	cypCar_00012555,LHQP01021640
*p40a*	*Interleukin-12 p40a subunit*	GAGCGCATCAACCTGACCAT	AGGATCGTGGATATGTGACCTCTAC	AJ621425
*p40b*	*Interleukin-12 p40b subunit*	TCTTGCACCGCAAGAAACTATG	TGCAGTTGATGAGACTAGAGTTTCG	AJ628699
*p40c*	*Interleukin-12 p40c subunit*	TGGTTGATAAGGTTCACCCTTCTC	TATCTGTTCTACAGGTCAGGGTAACG	AJ628700
*tnfαa1*	*Tumour necrosis factor α a1*	GAGCTTCACGAGGACTAATAGACAGT	CTGCGGTAAGGGCAGCAATC	AJ311800
*tnfαa2*	*Tumour necrosis factor α a2*	CGGCACGAGGAGAAACCGAGC	CATCGTTGTGTCTGTTAGTAAGTTC	AJ311801
*tnfαb1*	*Tumour necrosis factor α b1*	GAAGACGATGAAGATGATACCAT	AAGTGGTTTTCTCATCCTCAA	cypCar_00029601, LHQP01065580
*tnfαb2*	*Tumour necrosis factor α b2*	CTTGGACGAAGCCGATGAAGAC	ATCTTGTGACTGGCAAACA	cypCar_00023012, LHQP01037150
*tlr3.1*	*Toll-like receptor 3.1*	GTTATCCCTGGCGCATAATA	TCTTCAATAATTGGTAAGGATGATG	KF387571
*tlr3.2*	*Toll-like receptor 3.2*	GTTTATCCCTGGAGCATAACT	CTTCAATAACTGGTAAAGACGAAC	KF387572
*mx1*	*Interferon-induced GTP-binding protein Mx1*	ACAATTTGCGGTCTTTGAGA	CCCTGCCATTTCTCTTCG	cypCar_00015892, LHQP01004675
*mx2*	*Interferon-induced GTP-binding protein Mx2*	GCTTACGGTCTCTGGGG	TGGTTTCATCTTTAGTTCTTATCATC	cypCar_00029512, LHQP01026950
*mx3*	*Interferon-induced GTP-binding protein Mx3*	ACAAAGGACAATAACTGGCG	GAGGTCAGGAACATCACTG	cypCar_00017679, LHQP01012215
*mx4*	*Interferon-induced GTP-binding protein Mx4*	CTAGAGTTGCCACTGCC	TCCAGTTGAATCCACTTCG	cypCar_00025664, LHQP01010684
*mx5*	*Interferon-induced GTP-binding protein Mx5*	ACTGAAGTGTGTGTTTTTGG	CAGACCTGGTAGATAAAGGAG	cypCar_00012158, LHQP01006771
*gpr40La-1.1*	*G-coupled protein receptor 40-like subclass a 1.1*	GCCTTCTACACGCTCAG	CGCTCCACGCTCAC	MZ447840
*gpr40La-1.2*	*G-coupled protein receptor 40-like subclass a 1.2*	GAAGGCTGAGGGCG	AATAATCGGGTCCAAACAAG	MZ447841
*gpr40La-2*	*G-coupled protein receptor 40-like subclass a 2*	GTTTCCAATCCGATATGCT	TTATCACTTGAGGGTATGTATG	MZ447848
*gpr40Lb-1.1*	*G-coupled protein receptor 40-like subclass b 1.1*	TGTCATTTTCTCAGTGGTG	AAACGGCACGAGGAT	MZ447842
*gpr40Lb-1.2*	*G-coupled protein receptor 40-like subclass b 1.2*	GTTATGACGACTTCACTGAC	GATGGCTCTAAACCGCT	MZ447844
*gpr40Lb-2.1*	*G-coupled protein receptor 40-like subclass b 2.1*	TGCTCTTCCTCATACCG	GCTACATATCCAACCACG	MZ447843
*gpr40Lb-2.2*	*G-coupled protein receptor 40-like subclass b 2.2*	GCGGTGCTCTTCGTAAC	CCAGTCGGGGCTGTA	MZ447845
*gpr40Lc-1.1*	*G-coupled protein receptor 40-like subclass c 1.1*	CAACCCTTCCCAAAACA	ATTAAGAGCGGCAGCA	MZ447846
*gpr40Lc-1.2*	*G-coupled protein receptor 40-like subclass c 1.2*	GCCACCCTTCCCAAAAAT	GCAGCATGTATAGAACCAC	MZ447847
*gpr40Lc-2*	*G-coupled protein receptor 40-like subclass c 2*	TTCTGCTATATAAGCTGTATCTG	GAGACAAGTTGTGGGGT	MZ447849

Note: cypCar numbers identify ORFs in the common carp genome (BioProject: PRJNA73579) that were also confirmed by RNA sequencing. LHQP numbers refer to the accession number of the associated scaffold.

### Statistical Analyses

Statistical analyses were performed using SPSS (v23.0), and differences were considered significant if *p* ≤ 0.05. Data presented as fold change (gene expression analyses) were first transformed using natural log transformation [Y = LN(Y)] before analysis. All data were tested for Gaussian distribution using the Shapiro–Wilk test.

Differences in cumulative gas production were analysed with two-way ANOVA followed by the Bonferroni *post hoc* test. Differences in pH of the fermentation liquid after *in vitro* fermentation were assessed by Welch’s ANOVA followed by the Games–Howell test, as the data did not have equal variances. Differences in NH_3_ and short-chain fatty acid profiles in the fermentation liquid after *in vitro* fermentation were assessed by one-way ANOVA followed by Tukey *post hoc* test. Data for comparisons of NO production, ROS production, and gene expression *in vitro* were analysed using repeated measures and sometimes had some data points missing; therefore, analyses were performed with a linear mixed model, followed by a Bonferroni *post hoc* test. Comparison of gene expression *in vivo* was assessed by one-way ANOVA.

## Results

### Characterization of Intestinal Microbial Composition Implies β-Glucan Fermentation Capacity

Analysis of 16S ribosomal RNA rather than DNA provided insight into the active intestinal microbial communities. Identification of bacterial microbiota up to family level allowed us to investigate the presence of bacteria with theoretical fermenting capacities. The intestinal content of the third segment of the intestine from n = 5 individual fish was analysed by 16S rRNA sequencing, revealing a total of n = 55 active operational taxonomic units (OTUs) in the intestinal content of unhandled carp.

**Figure 1:**
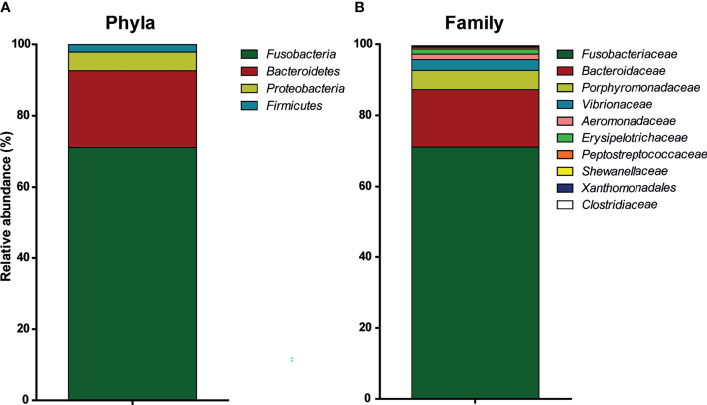
Relative abundance of active bacteria in the intestinal microbiota of common carp. Characterization with 16S rRNA sequencing of the intestinal microbiota of naive, unhandled common carp [(bars indicate relative abundance (mean, n = 5)]. **(A)** Relative abundance of phyla. **(B)** Relative abundance of family. *Xanthomonadales* refers to family *Xanthomonadales Incertae sedis*.

By far, the most represented phyla in the carp intestinal microbiota were *Fusobacteria* (71% ± 5.8% (mean ± SD, *n* = 5) of total reads), whereas *Bacteroidetes* (21% ± 5.3%), *Proteobacteria* (5% ± 0.5%), and *Firmicutes* (2% ± 0.5%) were considerably less frequent (**Figure 1A**). The majority of *Proteobacteria* belonged to the order of *Gammaproteobacteria* (95%), while the rest belonged to *Betaproteobacteria* (4%). Looking at the distribution per family in the active microbiota, the most abundant family were *Fusobacteriaceae* (71% ± 5.8% [mean ± SD, *n* = 5)], followed by *Bacteroidaceae* (16% ± 4%), *Porphyromonadaceae* (5% ± 2%), *Vibrionaceae* (3% ± 0.75%), *Aeromonadaceae* (1.5% ± 0.4%), and *Erysipelotrichaceae* (1.4% ± 0.1%) (**Figure 1B**). Finally, looking at the putative genus level, it becomes clear that all *Fusobacteriaceae* are member of the *Cetobacterium* genus, as 71% ± 5.8% (1 OTU [mean ± SD, *n* = 5)] of the total reads belong to this genus. Following *Cetobacterium* genus, the most abundant genera based on 16S RNA sequencing are *Bacteroides* (16% ± 4.1%, 2 OTUs), *Vibrio* (3% ± 0.75%, 1 OTU), *Aeromonas* (1.5% ± 0.4%, 1 OTU), and two different uncultured genera of the families *Porphyromonadaceae* (4.5% ± 4%, 3 OTUs) and *Erysipelotrichaceae* (1.1% ± 0.1%, 1 OTU). Although the sequencing effort could not identify bacteria at the species level, the abundant presence of the *Bacteroides* genus well known to express β-glucan-degrading genes (61) suggested the ability of carp microbiota to ferment β-glucans.

### *In Vitro* Batch Analysis Confirms Fermentation of β-Glucans

**Figure 2:**
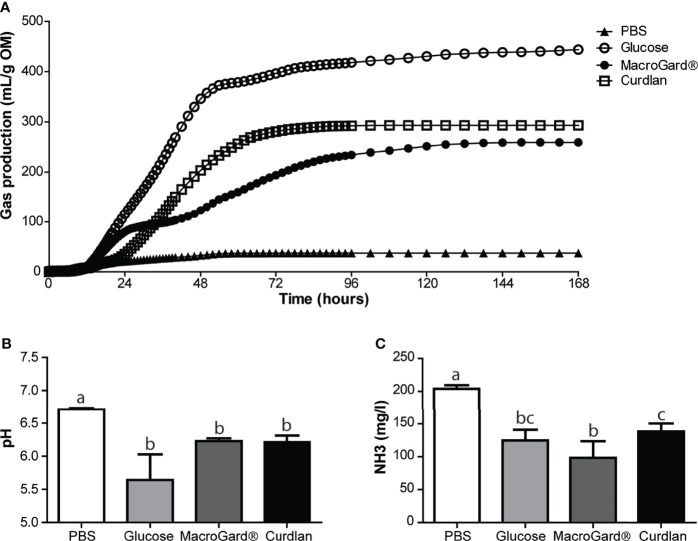
Carp naive microbiota is capable of *in vitro* fermentation of different β-glucans. **(A)** Cumulative gas production over 168 h as a result of *in vitro* fermentation of PBS, glucose, MacroGard^®^ and curdlan by carp intestinal microbiota. Data shown as mean of n = 5 independent intestinal pools. Significant difference between cumulative gas production between MacroGard^®^ and curdlan from 44–86 h [two-way ANOVA followed by Bonferroni *post hoc* (not shown)]. **(B)** pH of fermentation fluids after 168 h of *in vitro* fermentation (mean ± SD, n = 5). Significant differences between groups were assessed by Welch**’**s ANOVA followed by Games–Howell test. **(C)** Ammonia (NH_3_) accumulation in fermentation fluids after 168 h of *in vitro* fermentation (mean ± SD, n = 5). Significant differences between groups were assessed by one-way ANOVA followed by Tukey test. Groups with different letters are statistically different from one another.

In order to further investigate the ability of the naive intestinal microbiota of carp to ferment β-glucans, *in vitro* fermentation was performed (**Figure 2A**). Through an *in vitro* batch culture system, fermentation of different substrates could be analysed. As a measure for fermentation kinetics, cumulative gas production was measured over time. As a negative control, minor cumulative gas production was measured in the PBS group, similar to gas production in the negative control without substrate (data not shown). As a positive control, fermentation of the readily digestible monosaccharide glucose was measured, resulting in the highest cumulative gas production. Two different β-glucan preparations were studied: curdlan, a high molecular weight linear polymer consisting of β-1-3-linked glucose residues from *Alcaligenes faecalis*, and MacroGard^®^, a branched polymer β-(1,3/1,6)-glucose feed additive. In comparison, fermentation of curdlan resulted in the most continuous and highest cumulative gas production, while fermentation of MacroGard^®^ showed an intermediate plateau around 24–36 h. Gas production differed significantly between MacroGard^®^ and curdlan from 44 to 86 h after start of the *in vitro* fermentation (as assessed with a two-way ANOVA followed by Bonferroni *post hoc*, *p* ≤ 0.05). No matter the difference in kinetics of gas production between curdlan and MacroGard^®^, naive carp intestinal microbes were able to ferment β-glucans.

Finally, analysis of the fermentation liquid after *in vitro* fermentation revealed differences between substrates in pH and NH_3_, whereas these parameters did not differ at start (data not shown). In comparison, a (non-significant) trend toward lower pH was observed in the glucose group, compared to the two β-glucan-treated groups. No difference in pH was observed between MacroGard^®^ and curdlan treatment (**Figure 2B**). Ammonia production was significantly higher in the PBS group compared to all other groups (**Figure 2C**). Interestingly, NH_3_ production was different between curdlan and MacroGard^®^, providing further evidence for differences in fermentation between the two β-glucans.

### *In Vitro* Fermentation of β-Glucans Results in Significantly Increased SCFA Levels

**Figure 3:**
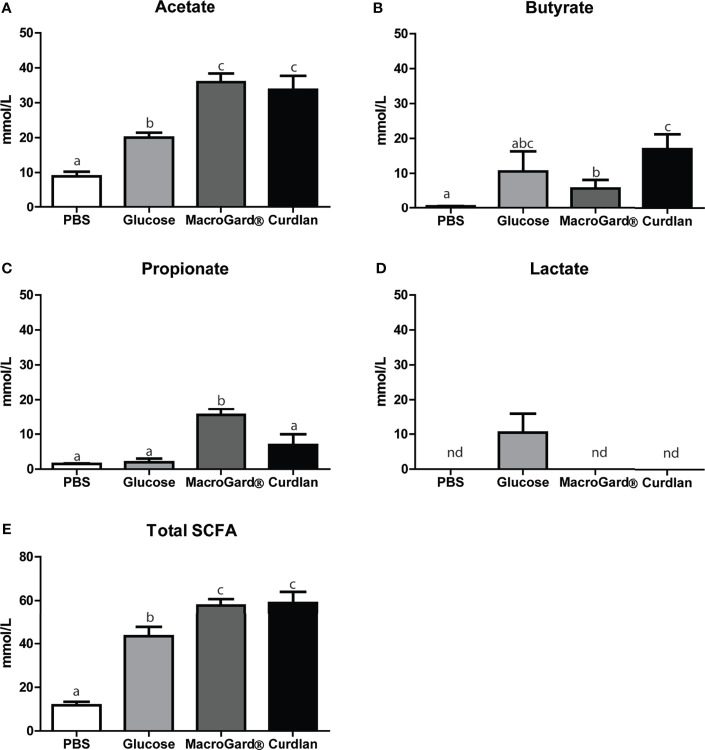
Differential SCFA profiles after *in vitro* fermentation of β-glucan. SCFA levels in the fermentation liquid following 168 h *in vitro* fermentation of PBS, glucose, MacroGard**^®^,** or curdlan. **(A–E)** Acetate, butyrate, propionate, and lactate, or total sum. Total SCFA levels were calculated by adding up all analysed SCFA levels including branched chain short-chain fatty acids. Bars indicate mean ± SD of n = 5 independent intestinal pools. Significant differences between groups were assessed by one-way ANOVA followed by Tukey test **(A, D, E)** or by Welch**’**s ANOVA followed by the Games–Howell test **(B, C)**. Groups with different letters are statistically different from one another. **(D)** The annotation “nd” indicates that lactate was not detected in these samples.

Results from the *in vitro* batch culture analysis provided evidence for the ability of intestinal microbiota of carp to ferment β-glucans, at least to a certain degree. In general, metabolites produced by microbiota during fermentation such as SCFA are often considered necessary components for immune homeostasis (62). We therefore measured the accumulation of SCFA metabolites in the *in vitro* fermentation liquid, produced during β-glucan fermentation. Significant differences in SCFA profiles between the readily fermentable substrate glucose and the two different β-glucan preparations could be observed (**Figures 3A–E**). Overall, a clear production of acetate, butyrate, and propionate was observed after fermenting either curdlan or MacroGard^®^ *in vitro*. Comparing the two different β-glucan preparations showed no differences in acetate production but revealed an interesting inverted trend for butyrate and propionate levels. After fermentation of curdlan, higher butyrate concentrations were measured, while after fermentation of MacroGard^®^ higher propionate concentrations were measured (**Figures 3B, C**). Profiles of other SCFA; formic, isobutyric, valeric, and iso-valeric acid were also measured, but concentrations were considered negligible (<0.1 mM, data not shown) and therefore excluded during the follow-up experiments. In both β-glucan groups, total SCFA levels were higher than in the glucose group; however, no differences were observed between both β-glucan groups (**Figure 3E**). Overall, differential production of SCFAs could be observed in both β-glucan groups, with fermentation of MacroGard^®^ specifically resulting in an increase in propionate. With the practical and commercial applicability of MacroGard^®^ being higher than of curdlan, our follow-up studies focused on the effects of fermentation of MacroGard^®^.

### Presence of SCFAs Modulates Radical Production by Head Kidney Leukocytes *In Vitro*

**Figure 4:**
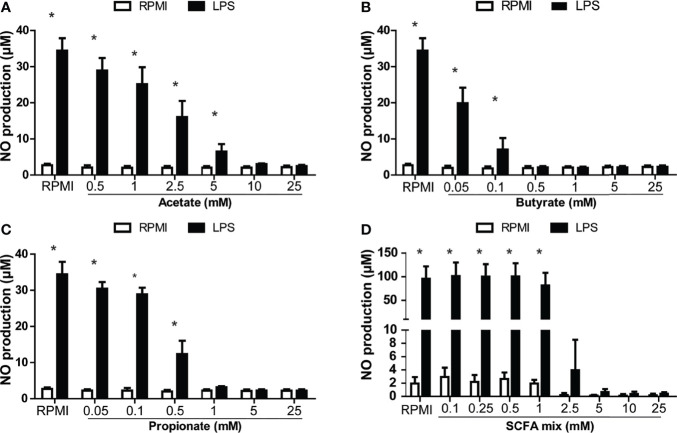
SCFA reduced LPS-induced production of nitric oxide (NO) in head kidney leukocytes (HKL). Cells were stimulated with medium control (RPMI) or LPS (30 µg/ml) for 96 h in the presence or absence of the SCFAs, acetate, butyrate, or propionate or a mix in the relative proportion produced by *in vitro* fermenting MacroGard^®^ (62.5%/10%/27.5%, acetate/butyrate/propionate). **(A)** NO production of LPS-stimulated HKL in the presence of acetate. **(B)** NO production of LPS-stimulated HKL in the presence of butyrate. **(C)** NO production of LPS-stimulated HKL in the presence of propionate. **(A–C)** Bars indicate mean ± SD of n = 3 independent experiments. **(D)** NO production of LPS-stimulated HKL in the presence of SCFA mix, indicating that the concentration is determined by the total amount of acetate, butyrate, and propionate (25 mM (15.625 mM acetate/2.5 mM butyrate/6.875 mM propionate). Bars indicate mean ± SD of n = 5 independent experiments. Note the difference in scale for the Y-axis in **(D)**. Asterisk (*) indicates significant difference between NO production induced in the presence (solid black bars) or absence of LPS (open white bars) assessed by the linear mixed model followed by the Bonferroni *post hoc* test.

In order to investigate the potential immunoregulatory effects of SCFAs, head kidney leukocytes (HKLs) were stimulated with LPS as a well-known stimulator of pro-inflammatory responses and stimulator of nitric oxide (NO) production or with zymosan as a well-known stimulator of pro-inflammatory responses and stimulator of ROS production in common carp, while different concentrations of SCFAs were added. Analysis of NO production at 96 h post stimulation revealed differential modulating potencies of the various SCFAs (**Figure 4**). Clear dose-dependent modulating effects could be observed for the three SCFAs investigated, but only in the presence of LPS. Butyrate was the most potent SCFA, followed by propionate and lastly acetate.

Furthermore, adding the SCFAs mixed in the same ratio as measured after *in vitro* fermentation of MacroGard^®^ (see **Figure 3**) showed similar reductions in *in vitro* induced NO production in HKLs (**Figure 4D**). Finally, titrating down this SCFA mixture revealed a significant reduction of LPS-induced NO production even at a concentration as low as 2.5 mM.

**Figure 5:**
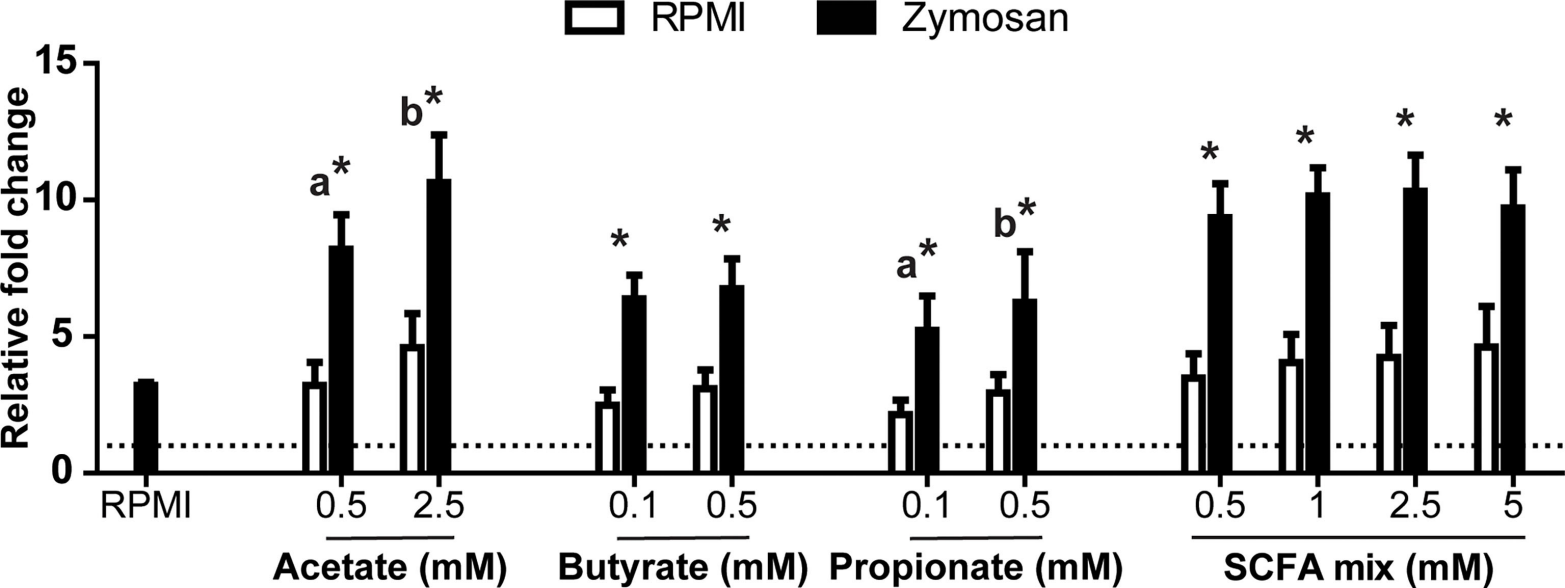
SCFA increased oxidative burst in head kidney leukocytes (HKL). Total production of reactive oxygen species (ROS) relative to unstimulated HKL (dotted line). Cells were exposed to different concentrations of SCFAs in the absence (medium control (RPMI), open bars) or presence (black bars) of zymosan (50 µg/ml). Immediately following exposure, ROS production was measured real-time over a period of 2 h. Bars indicate mean ± SD of n = 5 experiments performed independently. Asterisk (*) indicates significant difference between ROS production induced in the presence (solid black bars) or absence of Zymosan (open white bars) assessed by the linear mixed model followed by the Bonferroni *post hoc* test. Groups with different letters are statistically different from one another, within one treatment (acetate, butyrate, propionate or SCFA mix) where “a” indicates the lowest value.

Interestingly, when analysing the production of ROS, SCFAs appeared to increase the ROS production in a dose-dependent manner, although this trend was only significant for acetate and propionate (**Figure 5**). Furthermore, adding the SCFAs mixed in the same ratio as measured after *in vitro* fermentation of MacroGard^®^ (see **Figure 3**) showed similar modulations of *in vitro* production of ROS by HKLs. Both with and without the presence of a strong ROS inducer (zymosan), a clear increase could be observed in the ROS production of HKLs.

### Presence of SCFAs Modulates Cytokine and SCFA Receptor Gene Expression *In Vitro*

**Figure 6:**
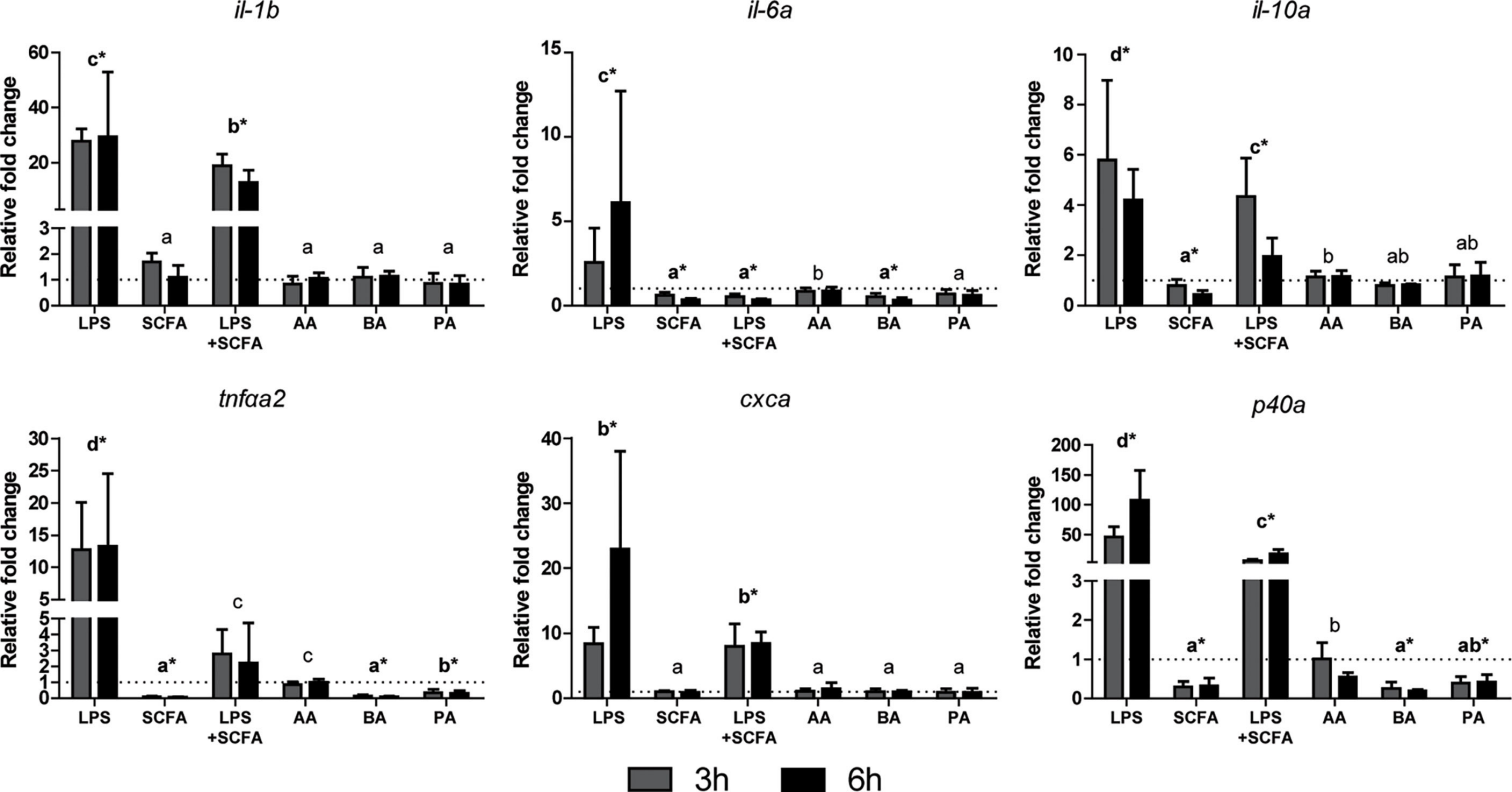
SCFAs reduce the expression of several cytokines *ex vivo* stimulated head kidney leukocytes. Gene expression after 3 (grey bars) and 6 (closed bars) h of stimulation with LPS (30 µg/ml), SCFA mix (2.5 mM), LPS, and SCFA mix or the three SCFAs at the concentration they were included in the mix; acetate 1.55 mM (AA), butyrate 0.25 mM (BA), and propionate 0.765 mM (PA), compared to unstimulated HKLs (dotted line). Bars indicate mean ± SD of n = 5 experiments performed independently. Significant differences between groups were assessed by the linear mixed model followed by the Bonferroni *post hoc* test. Groups with different letters are statistically different from one another where “a” indicates the lowest value, groups indicated with asterisk (*) are significantly different from unstimulated HKLs.

**Figure 7:**
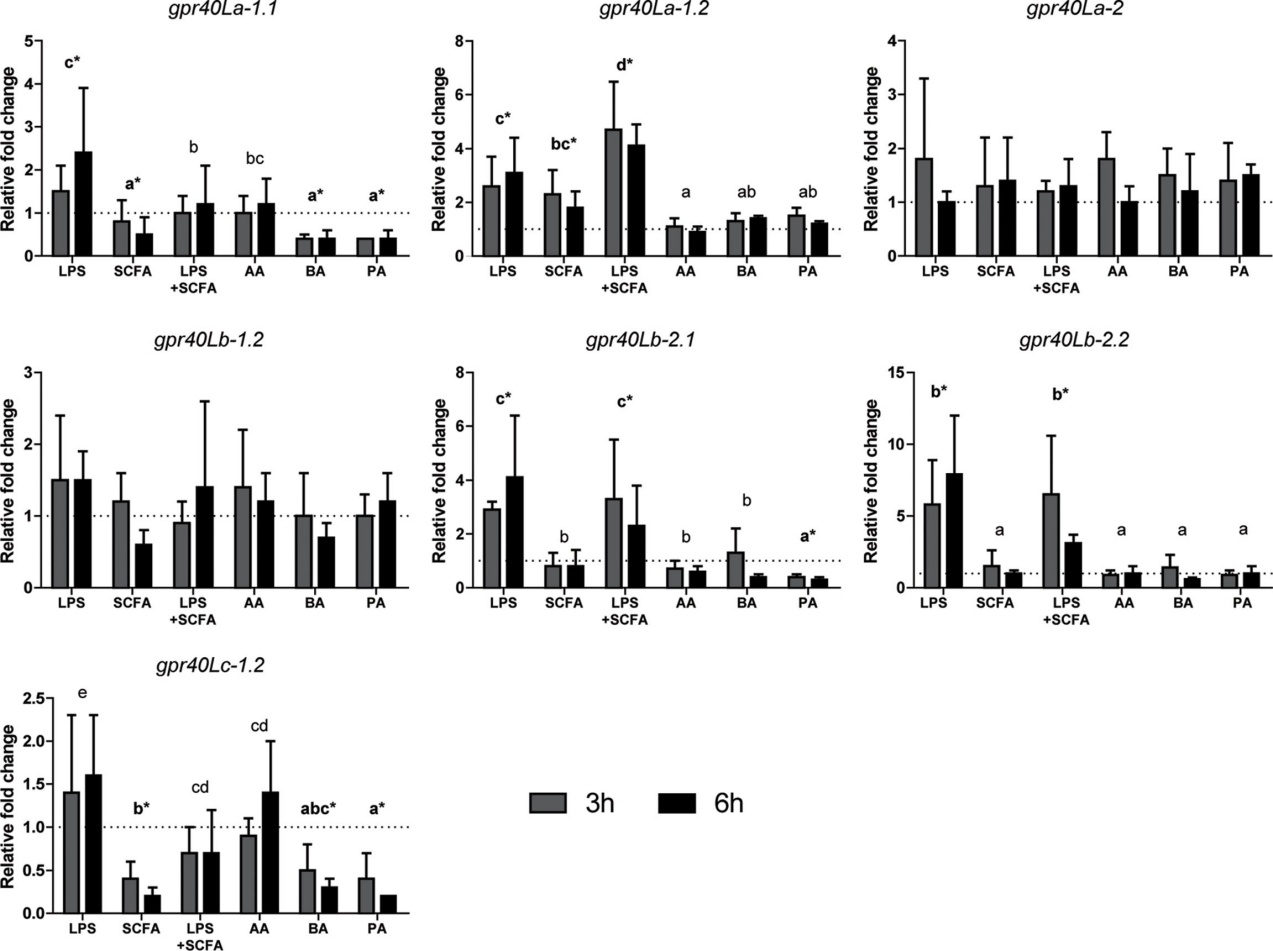
SCFAs regulate the expression of several *gpr40L* receptors *ex vivo* stimulated head kidney leukocytes. Gene expression after 3 (grey bars) and 6 (closed bars) h of stimulation with LPS (30 µg/ml), SCFA mix (2.5 mM), LPS, and SCFA mix or the three SCFAs at the concentration they were included in the mix; acetate 1.55 mM (AA), butyrate 0.25 mM (BA), and propionate 0.765 mM (PA), compared to unstimulated HKLs (dotted line). Bars indicate mean ± SD of n = 5 experiments performed independently. Significant differences between groups were assessed by the linear mixed model followed by the Bonferroni *post hoc* test. Groups with different letters are statistically different from one another, where “a” indicates the lowest value; groups indicated with asterisk (*) are significantly different from unstimulated HKLs.

Studying the gene expression can provide preliminary insights in the effects of SCFAs on inflammation, *via gpr40L* SCFA receptors. To this end, HKLs were stimulated *in vitro* with LPS, a mix of SCFAs in the same ratio as measured after *in vitro* fermentation of MacroGard^®^, both LPS and the mix, or with different SCFAs (acetate, butyrate, or propionate) separately. Subsequently, at 3 and 6 h post stimulation, cytokine gene expression (**Figure 6**) and *gpr40L* (**Figure 7**) gene expression were studied.

Addition of SCFAs generally resulted in downregulation of cytokine gene expression (**Figure 6**), either pro-inflammatory (*il-1b*, *il-6*, *tnfα*, *cxca*, *p40*) or anti-inflammatory (*il-10*), especially when first induced by presence of LPS. In the absence of LPS, addition of SCFAs resulted in a downregulation of cytokine gene expression of *il-6*, *tnfa*, and *p40*. Separately, SCFAs acetate, butyrate, and propionate showed slightly different levels of regulation, with acetate barely regulating gene expression. In general, addition of SCFAs as a mix showed comparable regulation as addition of SCFAs separately. Gene duplicates were also studied (*il-6b*, *il-10b*, *tnfαa1*, *tnfαb1*, *tnfαb2*, *cxcb*, *p40b*, and *p40c*, data not shown) and were regulated similarly to the genes presented in **Figure 6**).

Addition of SCFAs resulted in a differential regulation of gene expression of different *grp40L* receptors (**Figure 7**). Here, we studied gene expression of ten *gpr40L* genes and found three (*gpr40Lb-1.1*, *gpr40Lc-1.1*, and *gpr40Lc-2*) not expressed in HKLs. For the seven expressed gpr40L receptors, four were upregulated by stimulation with LPS (*grp40La-1.1*, *gpr40La-1.2*, *gpr40Lb-2.1*, *gpr40Lb-2.2*).

Regulation of the *gpr40L* expression in HKL by SCFAs was only stimulatory for *gpr40La-1.2* while inhibitory for *gpr40La-1.1*, *gpr40Lb-2.1*, and *gpr40Lc-1.2*. Again, of the different SCFAs analysed in this study, addition of acetate barely regulated gene expression.

### Oral Gavage With β-Glucans Modulates SCFA Receptor and Cytokine Gene Expression *In Vivo*

**Figure 8:**
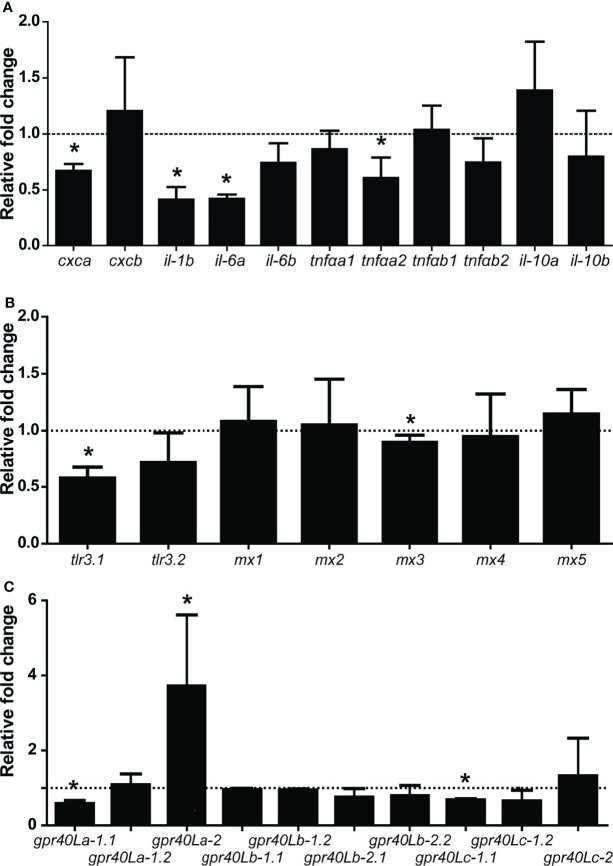
Oral gavage with β-glucans alters cytokine and gpr40L gene expression in the posterior intestine of carp. Gene expression of selected cytokines **(A)**, Type I IFN-related genes **(B)**, and *gpr40L* genes **(C)** in the posterior intestine of carp treated with the β-glucan group compared to the control group (dotted line), as measured by RT-qPCR 7 days post-gavage. **(A)** Significantly lower expressions of *cxca*, *il-1b*, *il-6a*, and *tnfαa2*. A trend toward a lower expression of *il-6b* (*p* = 0.062) and *tnfαb2* (*p* = 0.090) could be observed in the treated group. **(B)** Significantly lower expressions of *tlr3.1* and *mx3* **(C)** Significantly lower expressions of *gpr40La-1.1* and *gpr40Lc-1.1*, but significantly higher expression of *gpr40La-2*. Bars indicate mean ± SD of n = 5 animals. Asterisk (*) indicates significant difference in expression between the group orally gavaged with PBS and the group orally gavaged with 1 mg MacroGard^®^ as assessed by one-way ANOVA.

To follow up on the above-described *in vitro* modulating effects of SCFAs, we subsequently analysed the *in vivo* gene expression locally in the posterior intestine in response to a single oral gavage with β-glucans (MacroGard^®^). Based on the *in vitro* fermentation results (see **Figures 2**, **3**) and the assumption that also *in vivo* the active naive microbiota of common carp can ferment β-glucans and lead to the production of specific SCFAs, we measured whether the effects of an oral gavage with β-glucans could be measured by a change in gene expression of *gpr40L* and other genes. Analysis of microbiota composition on day 7 and day 14 showed no significant differences between the control (PBS) and the β-glucan-treated group (data not shown). Gene expression regulation was most clear at 7 days post gavage (**Figure 8**), while gene expression profiles on 3, 11, and 14 days post gavage did not show distinct trends (data not shown). Similar to the *in vitro* observations on HKL, we found a significant downregulation in the posterior intestine of *cxca*, *il-1b*, *il-6*, and *tnfa* expression at 7 days post gavage (**Figure 8A**). Analysis of the expression response of type I IFN-related genes (*tlr3.1*, *tlr3.2*, and *mx1-5*) revealed a clear downregulation of *tlr3.1* and a marginal but significant downregulation of *mx3* expression (**Figure 8B**). Further, both *in vitro* and *in vivo gpr40La-1.1* gene expression was downregulated (**Figure 8C**), but differently, *gpr40La-2* expression was significantly upregulated only *in vivo*. Of interest, all ten *gpr40L* receptors were expressed in the posterior intestine.

## Discussion

To investigate whether dietary β-glucans can alter the composition or activity of the intestinal microbiota, we first characterised the active intestinal microbiota of unhandled carp or naive intestinal microbiota. We observed, among others, a prominent presence of the genus *Bacteroides*, of interest because some species of this genus have been shown able to degrade β-glucans (61). Subsequently, we confirmed that the carp naive intestinal microbiota has β-glucan fermenting ability by using an *in vitro* batch culture system to test fermentation of two different β-glucan preparations (curdlan and MacroGard^®^). The *in vitro* fermentation of these two β-glucan preparations led to the production of several and different short-chain fatty acids (SCFAs), of which an increase in propionate could be specifically linked to the fermentation of MacroGard^®^. We first confirmed the hypothesis that SCFAs might have immunomodulatory activity by titrating different SCFAs on head kidney leukocytes *in vitro*, measuring the modulation of induction of NO production and oxidative burst capacity. We also measured the immunomodulatory activity of SCFAs by determining gene expression and could show a clear and mostly downregulation of cytokine expression and putative SCFA receptor *gpr40L* genes. *In vivo*, administration of β-glucans by oral gavage with MacroGard^®^ also modulated the gene expression of several cytokines and *gpr40L* genes in the posterior intestine. Of particular interest for follow-up studies could be the upregulation of one particular *gpr40L* receptor sub-variant (*gpr40La-2*). Collectively, our data provide support for the hypotheses that β-glucans can act as substrate for fermentation by intestinal microbiota of common carp, thereby possibly inducing an alteration of local intestinal microbial composition and activity with associated changes in production of SCFA. Altered ratios of SCFA could then be sensed by *gpr40L* receptor(s) and following downstream signalling, modulating immune responses. Such a line of events could provide a logical explanation for the modulation of immune responses by β-glucans, at least in common carp.

We identified four abundant phyla in the naive intestinal microbiota of common carp (*Fusobacteria*, *Bacteroidetes*, *Gammaproteobacteria*, *Firmicutes*) using 16S rRNA analysis. Of the phylum *Fusobacteria*, the genus *Cetobacterium* clearly dominated in abundance and appears to do so in omnivorous and carnivorous species more so than in herbivorous fishes (63). The genus *Bacteroides* appeared as the second most abundant and is particularly interesting for its linkage to fermentation of carbohydrates and capability of degrading even highly complex carbohydrates (64–66). Degradation of carbohydrates is achieved with so-called polysaccharide utilization loci (PUL) of which there can be many different ones, each related to the degradation of a specific glycan or group of related glycans (67). At least in humans, members of the *Bacteroides* found in the gastrointestinal tract express such PUL and have been shown able to degrade several different β-glucans, among which barley-derived β-1,4-glucans (65) and fungus-derived β-1,6-glucans (61). Interestingly, analysis of microbiota composition following oral gavage with β-glucans *in vivo* revealed no alteration of the composition, arguing that a single oral gavage with β-glucans will not alter the composition, but can alter the metabolism. In contrast to other studies investigating the effects of β-glucans on fish microbiota composition, those studies investigated the effect of constant exposure during dietary supplementation (27–33). Our microbiota sequencing data confirm the presence of bacterial genera commonly associated with β-glucan-fermenting capacity. Future metagenomic approaches are needed to confirm and study in more detail the presence of bacterial species with β-glucan-fermenting ability in the naive microbiota of carp.

Ours is not the first study suggesting that fish microbiota could have the ability to degrade carbohydrates; others already observed fermentation of several carbohydrates and production of SCFAs by intestinal microbes of tilapia, European sea bass, and common carp (23, 25), or showed growth of fish intestinal bacteria on different carbohydrate sources, including β-glucan (22). To verify the β-glucan fermenting capacity of naive carp intestinal microbiota, we performed an *in vitro* batch culture experiment using two different β-glucans as substrates: MacroGard^®^, a branched β-1,3/1,6-glucan isolated from the yeast *Saccharomyces cerevisiae* and commonly used in aquaculture practices, and curdlan, a linear β-1,3-glucan isolated from the bacteria *Alcaligenes faecalis*. Analysis of gas production after fermentation of both β-glucans revealed distinct fermentation curves. Gas production differed significantly between the two β-glucan substrates in the period of 44–86 h after the start of fermentation. Fermentation also led to specific SCFA profiles, with higher butyrate production associated with curdlan and higher propionate production associated with MacroGard^®^. It cannot be fully excluded that some of the differences in gas production and SCFA profiles between the two β-glucan substrates might be caused by “contamination” of MacroGard^®^ with products such as chitin and mannose (10). Mannose can inhibit carbohydrate uptake of bacteria under specific conditions (68), and chitin can also be fermented, which could muddle SCFA profiles, since, at least in tilapia, chitin is fermented in acetate and propionate but not in butyrate (23). Regardless of these considerations, *in vitro* fermentation of β-glucans by the intestinal microbiota of carp resulted in increased gas and in the production of SCFA, especially acetate, butyrate, and propionate.

Also in mammals, the main SCFAs produced as metabolites of fermentation of resistant starches, fibre, and polysaccharides are acetate, butyrate, and propionate. Butyrate and propionate have been associated with clear immunomodulatory effects such as cancer treatment in humans (69). Further, butyrate and propionate are known to regulate inflammatory responses in epithelial cells and leukocytes (70–74). These encompass, but are not restricted to, effects on leukocyte recruitment, cytokine production, lymphocyte activation, or even phagocytosis or oxygen radical production [as reviewed by (75)]. Interestingly, there are also studies which show promotion of inflammatory responses *via* SCFAs (76, 77). Within an immunological context, butyrate and propionate are currently considered the two most potent SCFAs [as reviewed by (77)]. For example, both have been shown in humans to inhibit LPS-induced TNF-α production *via* inhibition of NF-κB and increased IL-10 production (78, 79) or to inhibit antigen-specific cytotoxic T-lymphocyte activation *via* the regulation of IL-12 production in dendritic cells (DCs) (80). Several studies in mice have connected oral intake of fibres to increased SCFA levels, both local and systemic, and linked these increased SCFA levels with subsequent reductions in pathogenesis. For example, mice fed with oat bran-derived β-glucan-enriched diets had higher SCFA levels in the cecum, especially after having been fed diets with practically insoluble β-glucan of heavy molecular weight (81). Further, mice fed with high-fibre diets showed systemic increases in propionate levels, while oral supplementation with propionate reduced allergy-induced inflammation in the lungs (82). Often, such studies highlight important signalling roles for SCFAs receptors such as the G-protein-coupled receptors GPR41 and GPR43 (also known as free fatty acid receptor 2 or FFAR2) (73), and to a lesser extent for GPR109A and OLFR78, or mention SCFA mediation *via* their effect on histone deacetylases (HDACs) [as reviewed by (77)]. Up to date, for fish a direct link between fermentation of immunomodulating products such as β-glucans, and production of SCFAs with subsequent reduction of pathogenesis has not been clearly shown. Nevertheless, over the past years, incorporation of SCFA-related products in fish feed has received increasing interest (83). Several studies have investigated the effects of SCFA salts on several immune parameters in fish. For example, incorporation of sodium propionate into the diets of zebrafish for 8 weeks induced expression of *il-1b* and *tnfα* (84). Further, supplementation of the diet of common carp with encapsulated sodium butyrate affected *il-1b*, *tnfα*, and *tgf-β* expression after feeding for 8 weeks (85). Also, incorporation of calcium propionate or calcium lactate in the diet of Nile tilapia increased fish performance and affected haematological parameters (86). Last but not least, a recent study in zebrafish showed that immersion of zebrafish larvae in butyrate reduced migration to a wound area of both neutrophils and pro-inflammatory macrophages, while specific knockdown of hydroxycarboxylic acid receptor 1 (*hcar1*) abolished the reduced migration of the neutrophils (87). The study in zebrafish will support further research into the immunomodulating role of SCFAs, not only in this model animal but also in fish species of commercial interest.

Recently, we used genomic resources and cDNA cloning to identify and validate ten coding sequences for *gpr40L* genes in common carp (37). Phylogenetic analysis showed a division into three subclasses and a closer relationship to mammalian GPR43 than to mammalian GPR41. Synteny analysis revealed a clear conservation of syntenic relationships between carp *gpr40L* and the relevant region in the human genome. Here, we provide a first but important step toward a further understanding of the role and function of these *gpr40L* receptors and link with immunomodulation in fish. In our study, leukocytes isolated from a primary lymphoid organ (head kidney derived) expressed seven out of ten *gpr40L* receptors; the expression of some but not all could be stimulated by LPS, or inhibited by SCFA. Expression of *gpr40L* receptors may differ between leukocytes from different organs and may change with maturation stage; therefore, in our analysis leukocytes from a primary lymphoid organ were used. The posterior intestine expressed all ten *gpr40L* receptors, but expression of only one particular SCFA receptor (*gpr40La-2*) was upregulated after oral gavage with β-glucans. It remains to be proven whether modulation of the gene expression of specific *gpr40L* receptors in the fish intestine could be a reliable read-out for immunomodulating effects of products such as β-glucans.

Although we hypothesised that the change in SCFA profile associated with fermentation of β-glucans and subsequent regulation of *gpr40L* as SCFA receptors might explain the downregulation of cytokine gene expression observed in the carp intestine, there might be alternative explanations. For example, in mice, commensal lactic acid bacteria can induce an antiviral IFN response induced by recognition of shedded, double-stranded RNA by intestinal DCs. Sensing *via* a TLR3*-*mediated mechanism then leads to the production of IFNβ, which in turn dampens the expression of inflammatory cytokines such as *Il-6* and *Tnfα* (88). Of interest, in a previous study we described a concurrent increase in *mx* and *tlr3* expression in the intestine of carp fed with β-glucans for 25 days, and clear downregulation of *il-1b*, *il-10*, and *tnfα* (89). In our present study, we observed a similar inhibition of *il-1b*, *il-6*, and *tnfα* in carp intestine but could not repeat all the observations by Falco and colleagues; i.e., a downregulation of *tlr3.1* and *mx3* was observed in the current study in contrast to the significant upregulation of *tlr3* and *mx* by Falco and colleagues. This leaves the presence of dsRNA as an alternative explanation for the downregulation of cytokine gene expression in the intestine to be confirmed. No matter the presence of dsRNA and/or altered ratios of SCFA detected by their receptors, regulation of pro-inflammatory gene expression in the fish intestine by supplementation with β-glucans was evident.

In the future, it can be of interest to analyse immunomodulatory effects of β-glucans on sterile intestinal samples, for example using *ex vivo* stimulation of gut tissue, to further investigate the contribution of the active naive microbiota to β-glucan-induced regulation of immune gene expression. Such an experimental setup could also allow for selectively testing immunomodulatory effects of SCFAs and other microbial metabolites. The present study provides support for a line of reasoning that suggests β-glucans can be fermented by intestinal microbiota and lead to changes in production of SCFA. Certainly, investigating the role of *gpr40L* receptor(s) in such processes will be of great interest.

## Data Availability Statement

The datasets presented in this study can be found in online repositories. The names of the repository/repositories and accession number(s) can be found as follows: https://www.ncbi.nlm.nih.gov/, PRJNA766179.

## Ethics Statement

All animals were handled in accordance with good animal practice as defined by the European Union guidelines for the handling of laboratory animals (http://ec.europa.eu/environment/chemicals/lab_animals/home_en.htm), local experimental animal committee (DEC number 2015098).

## Author Contributions

JP, IdB, WP, EvdB, MF, and GW contributed to the design of the experiments. JP, MG, and EvdB contributed to the acquisition of samples and analysis of data. GW acquired funding. WP contributed to the *in vitro* digestion analysis. IdB contributed to the analysis of microbiota. JP, IdB, MG, WP, MF, and GW wrote the manuscript. All authors contributed to the article and approved the submitted version.

## Funding

Research leading to this review was funded by the Netherlands Organisation for Scientific Research and São Paulo Research Foundation, Brazil (FAPESP), as part of the Joint Research Projects BioBased Economy NWO-FAPESP Programme (project number 729.004.002).

## Conflict of Interest

The authors declare that the research was conducted in the absence of any commercial or financial relationships that could be construed as a potential conflict of interest.

## Publisher’s Note

All claims expressed in this article are solely those of the authors and do not necessarily represent those of their affiliated organizations, or those of the publisher, the editors and the reviewers. Any product that may be evaluated in this article, or claim that may be made by its manufacturer, is not guaranteed or endorsed by the publisher.

## Footnotes

1
https://github.com/nioo-knaw/hydra/tree/1.3.3
